# Does Theory of Mind Training Enhance Empathy in Autism?

**DOI:** 10.1007/s10803-018-3671-1

**Published:** 2018-08-03

**Authors:** Annaleena Holopainen, Daniëlle M. J. de Veld, Elske Hoddenbach, Sander Begeer

**Affiliations:** Section Clinical Developmental Psychology, Vrije Universiteit Amsterdam, Van der Boechorststraat 1, 1081 BT Amsterdam, The Netherlands

**Keywords:** Autism spectrum disorder, Empathic responsiveness, Theory of mind, Intervention, Randomized controlled trial

## Abstract

Youth with ASD often show limited or atypical empathic responsiveness. The direct effects of social skills interventions on enhancing empathic responsiveness is unknown. Data from a randomized controlled trial were used to investigate whether a Theory of Mind training improves the empathic responsiveness, measured through structured observations. The current study included a large sample (n = 135) of 8–13-year-old children with ASD. When comparing the change scores of empathic responsiveness from baseline to post-test, the intervention group performed significantly better than the waitlist group. Thus, the current findings support the use of Theory of Mind training as intervention of ASD by showing its efficacy also in improving one’s empathic responsiveness, in addition to previous knowledge regarding the improvements in empathic understanding.

## Introduction

The ability to empathize is crucial for social functioning and wellbeing (De Waal 2008; Fink et al. 2014; Gaudion et al. 2014); while a lack of empathy is often considered one of the core social deficits in autism spectrum disorder (ASD) (Baron-Cohen and Wheelwright 2004). Various social skills interventions have been developed to target the social deficits present in ASD (Gates et al. 2017). One such intervention is Theory of Mind training (ToM), which is efficacious in improving children’s empathic understanding (*Theory of Mind*) and parent-reported social understanding (Begeer et al. 2011, 2015). Yet, its effect on objectively measured real-life empathic responsiveness is currently unknown. Therefore, we investigated whether the ToM training improves empathic responsiveness in children with ASD, as assessed through structured observations.

The concept of empathy can be divided into two domains: the social-cognitive understanding of other people’s mental states (i.e. *Theory of Mind*) and the more behavioural aspect, namely *empathic responsiveness* (Baron-Cohen 2000; De Waal 2008; Lawrence et al. 2004). Trials on ToM training have shown that interventions can improve both children’s ToM understanding and their ToM-related behaviour as assessed with child-based measures and parental reports (Begeer et al. 2011, 2015; Fletcher-Watson et al. 2014). However, questionnaires may be misinterpreted or insensitive to change; while optimal assessment conditions may also be very different from real-life situations (Begeer et al. 2010, 2015). Furthermore, parents observe their child’s behaviour in different contexts over time, which may actually limit their ability to notice the most recent changes (Scheeren et al. 2013). These limitations to previously used measures, combined with the crucial role of real-life empathic behaviour for one’s social functioning (Fink et al. 2014), indicate that it is important to supplement the currently used test battery to study ToM training efficacy with more ecologically valid measures.

Empathic capabilities can be measured with structured observation, which refers to observing a person’s empathic responsiveness to another person’s expressed emotion (Newbigin et al. 2016; McDonald et al. 2017; Scheeren et al. 2013). Assessing behaviour with a structured observation may overcome the limitations of questionnaires (Scheeren et al. 2013). For instance, when compared to a parental report, structured observation may lower the risk of external factors influencing the assessment (e.g. recent situation in a family, one’s role in a social group). Importantly, empathic behaviour is not always expressed verbally, thus structured observations ought to assess both verbal and non-verbal reactions (Scheeren et al. 2013).

The aim of this study was to investigate whether the ToM training improved empathic responsiveness of youth with ASD when assessed with a structured observation. Children were observed at baseline and post-test in two different situations that both warranted an empathic response. A previous sub-study of the current trial (Begeer et al. 2015) found an intervention effect on children’s ToM-related behaviour measured by parental reports. Therefore, we similarly hypothesized there to be an intervention effect on empathic responsiveness as assessed through structured observations.

## Method

### Participants

The study sample included 135 children with ASD, of whom 119 were boys. At the first meeting the children were 8–13 years old (*M* = 9.5, *SD* = 1.67). They were randomly assigned either to an intervention group (72 children) or a waitlist control group (63 children). Inclusion criteria were a clinical diagnosis of ASD according to DSM-IV-TR (APA 2001) and a verbal IQ score within the normal range (> 70) based on the Peabody Picture Vocabulary Test-III-NL (PPVT) (Dunn et al. 2005). Diagnostic procedure included multiple assessments by both a psychologist and a psychiatrist, who were not involved in the current study. Importantly, regardless of the clinical diagnoses, 9.2% of the participants (n = 12) scored below the clinical significance (≥ 59) on the Social Responsiveness Scale (SRS). Participants were recruited from De Bascule, an academic centre in Amsterdam focused on child and adolescent psychiatry. The study was approved by the VU University of Amsterdam Human Ethics Committee. Informed consent was given only by the parents, as children in the current study were younger than 16 years, and did not have to provide active informed consent in the Netherlands (See Table 1 for participant details).

**Table 1:** Demographic information of intervention and waitlist groups at the baseline (n = 135)

Group	Intervention (n = 72)	Control (n = 63)	*p* (*t* test or χ^2^)
Age (year)	Mean: 9.6 (SD: 1.65) [range 8–12]	Mean: 9.4 (SD: 1.7) [range 8–13]	*t* test: − 0.636, *p* = .526
Gender			χ^2^: 0.062, *p* = .0803
Male	63 (87.5%)	56 (88.9%)	
Female	9 (12.5%)	7 (11.1%)	
PPVT	Mean: 108.7 (SD: 14.3) [range 80–149]	Mean: 106.5 (SD: 12.1) [range 77–136]	*t* test: − 0.955, *p* = .342
SRS	82.0 (SD: 21.9) [range 34–138]	Mean: 83.1 (SD: 20.4) [range 39–123]	*t* test: 0.306, *p* = .76
Empathic responsiveness (ER)	3.34 (SD: 0.79) [range 1–4]	3.5 (SD: 0.72) [range 1–4]	*t* test: 0.559, *p* = .577
ER ≤ 2	8 (9.8%)	6 (10%)	
2 < ER ≤ 3	16 (23.4%)	15 (22.9%)	
ER > 3	48 (66.7%)	42 (67.2%)	
Diagnosis			χ^2^: 3.58, *p* = .466
PDDNOS	47 (65.3%)	42 (66.7%)	
Asperger	21 (29.2%)	13 (20.6%)	
Autism	2 (2.8%)	6 (9.5%)	
Comorbidity			χ^2^: 5.0, *p* = .758
None	46 (63.9%)	42 (66.7%)	
ADHD	20 (27.8%)	14 (22.2%)	
ADD	2 (2.8%)	2 (3.2%)	

### Intervention

The shortened version of ToM training (‘Mini ToM Intervention’) included 8 weekly sessions of 1-hour each (Begeer et al. 2015). The sessions always had the same structure: discussion, exercises, summarising the meeting for parents, and presenting a new homework. The sessions focused on ToM-related topics, such as emotion recognition, pretence, false belief, and humour. The training was delivered in a psychiatric centre, for five or six children simultaneously. In the groups the mutual age difference did not exceed 3 years. Sessions were supervised by a certified clinician who had received training for this manualized intervention. Additionally, parents attended two sessions where they were informed about the ToM intervention. During these two sessions, the parents also had a possibility to discuss how they could support their children to further improve the gained social skills. The intervention is described in more detail in the trial protocol (Hoddenbach et al. 2012).

### Measures

*Structured observations of empathic responsiveness* consisted of situations where an experimenter expressed either excitement or surprise. Both situations, excitement and surprise, were measured both pre- and post-test. Excitement was expressed by saying “I’m really looking forward to tomorrow!” or “I’m really looking forward to next week!”. Regarding surprise, the experimenter suddenly looked pass the child and said “Huh?!”, as if noticing something in the corner of the room. These emotional expressions resembled natural situations and they were placed between tasks that were part of large battery of psychological tests. We assumed that surprise and excitement would be appropriate prompts to elicit empathic responsiveness, because they trigger the child to consider the underlying beliefs or desires of these emotions. Furthermore, these two situations were chosen, because they could be easily embedded in the brief break in between other psychological assessments, and because an experimenter could easily express them without reliance on acting skills. Similar methods have also been used in previous studies (Scambler et al. 2007; Scheeren et al. 2013; Newbigin et al. 2016). Reactions of the participants were video-recorded and two independent research assistants coded them into 14 nominal categories, based on verbal and non-verbal behaviour. The raters were blinded to the condition and timepoint. Average scores of the two raters were used in the analyses. The interrater reliability between the two independent assistants reached a sufficient agreement, kappa ranging from 0.835 to 0.922. Before analyses, these scorings were recoded into one dependent variable with five ordinal categories, namely (1) empathic response, (2) relevant response, (3) confirmatory response, (4) attention without response, and (5) irrelevant response. These categories are presented in Table 2. Additionally, Table 3 presents how the original nominal categories were recoded to the five ordinal categories.

**Table 2:** Categories of responses obtained from structured observations

Category	Definition	Examples of responses to other’s emotional states	
		Excitement (e.g. “I am looking forward to tomorrow!”)	Surprise (e.g. “Huh?”)
Empathic response	Child gives a relevant verbal response including an empathic reference to the other’s emotional state, or offers solutions to alleviate the other’s distress	- “That sounds like fun.” - “That’s nice.”	- “Is something wrong?” - “Do you see something hideous?”
Relevant response	Child gives a relevant verbal response, but response does not include an empathic reference to the other’s emotional state or solutions to alleviate the other’s distress	- Where are you going? - Why?	- “What do you see?”
Confirmatory response	Child briefly confirms that he/she has heard the other person	- Nodding, smiling - “Ok,” “Yes”	- Nodding - “Ok”
Attention without response	Child attends to the other person, but does not give a response	- Looking, but no response	- Looking, but no response
No response or irrelevant response	Child does not attend or respond to the other person, or gives an irrelevant or inappropriate response	- No attention or response - “Do you have a scale?”	- No attention or response - “I did not have any honey last time, that tasted good.”

**Table 3:** Ordinal scoring (1–5) and its relation to the original nominal scoring (0–13)

Score 1–5	Definition	Score 0–13	Definition
5	Empathic response	9	Relevant empathic verbal response
4	Relevant response	6	Relevant, specific verbal response, focused on the child himself
		7	Relevant, specific verbal response, answer aimed at researcher
		8	Relevant verbal response, aimed at a solution
		12	Relevant verbal response
		13	Relevant verbal response, asks question directed to researcher
3	Confirmatory response	5	Relevant reaction, but not verbal
		11	Verbal confirmation
2	Attention without response	4	Attention, but no response
1	No response or irrelevant response	0	Other
		1	Inappropriate response
		2	No attention, no response
		3	Irrelevant verbal response
		10	Uncomfortable response

Verbal intelligence of the participants was assessed with *The Peabody Picture Vocabulary Test* (PPVT) (Dunn et al. 2005). The PPVT has a high internal consistency (0.92–0.98) and test–retest reliability (0.91–0.94), and additionally it correlates highly with the WISC-III verbal IQ (Hodapp and Gerken 1999).

The *Social responsiveness Scale* (SRS) (Constantino and Gruber 2007) was used to assess autistic features. The SRS is a parental report with a 4-point Likert scale (i.e. 0 = never true, 4 = almost always true). A higher score in SRS refers to more autistic features. Previous research has shown SRS to have a good internal consistency (0.91–0.97), test–retest reliability (0.84–0.87) and interrater reliability (0.76–0.95) (Bölte et al. 2008).

The ToM Behavior Checklist (ToMbc) (Begeer et al. 2015) was used to assess a child’s ToM-related behaviour in everyday life. This parent report assesses the frequency of ToM-related behaviour across eight domains of behaviour on a 6-point Likert-scale (0 = never, 5 = very often). A higher score indicates thus a higher frequency of ToM-related behaviour. A previous study has shown the ToMbc to have a good reliability (0.81) (Begeer et al. 2015).

### Procedure

**Fig. 1:**
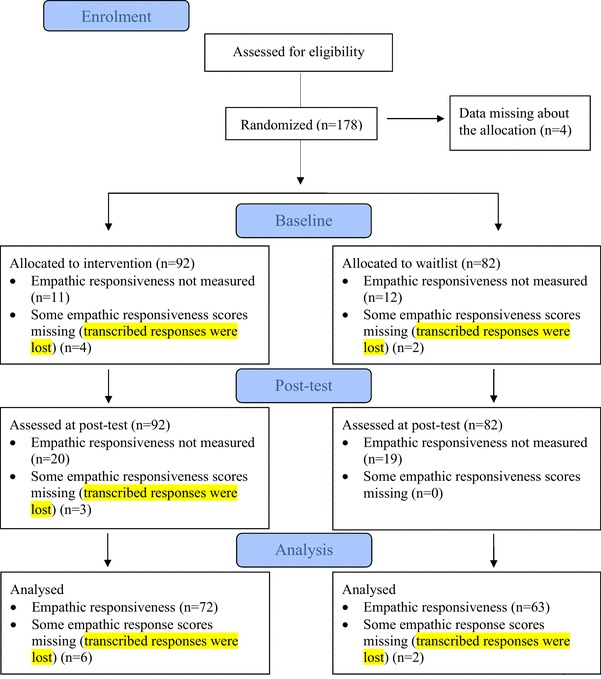
CONSORT 2010 flow diagram

A digital random number generator was used to randomly assign participants to an intervention or waitlist condition. An independent researcher conducted the randomisation and the primary investigator informed the parents whether their child was assigned to the intervention or waitlist. For the intervention group, the baseline assessment was done right before intervention and the post-test assessment was done right after the intervention ended (8 weeks after baseline). For the waitlist group, baseline assessment was done 8 weeks prior to the intervention and post-test assessment right before intervention. The participant flow is presented in more detail in Fig. 1.

### Analyses

The analysed sample consisted only of participants who were included to the assessment of empathic responsiveness (n = 135 out of the original 178 participants). Some of the included participants had missing scores. These were replaced with a value of the other situation (i.e. excitement or surprise), measured at the same assessment point. Thus, if only one score (i.e. one situation) was missing at baseline, it was replaced with the existing score (i.e. other situations) at baseline. The same was done for post-assessment. In total only six participants missed one score at the baseline and three participants at the post-test. If scores for both situations were missing at baseline or at post-assessment, participant was excluded from the analysis. Importantly, verbal intelligence of the included sample, measured with the Peabody picture Vocabulary Test, was significantly higher compared to the excluded sample (*p* < .05).

First, to gather more information regarding the sensitivity of the structured observations, we analysed the variation in empathic responsiveness. This was done by calculating the number of participants in each of the five categories at the baseline. Second, the two groups were compared based on the demographic information with the *t* test or χ^2^-test to ensure a successful randomisation (Table 1). Third, the efficacy of the intervention on empathic responsiveness was tested with the repeated measures analysis of variance (ANOVA). Time was added to the model as a within-subject factor and intervention condition as a between-subject factor. Additionally, an exploratory analysis was conducted to test whether the change scores differ between the two assessment situations (i.e. excitement and surprise). Here, time and two different assessment situations were added as within-subject factors and treatment condition as a between-subject factor. The latter analysis was run with a slightly smaller sample due to some missing values.

## Results

Prior to analysis, we first ensured that the randomisation was successful. Analyses indicated that the intervention and control group were comparable in terms of age, verbal ability, autism severity, baseline empathic ability, gender, diagnosis, and comorbidity (see Table 1). Second, we tested the correlations between empathic responsiveness across the two situations. The correlations between excitement and surprise were significant, although not very strong, at baseline (T1: *r* = 24, *p* < .01) and at post-test (T2: *r* = .19, p < .05). The correspondence over time in the two situations was stronger (excitement *r* = .25, *p* < .01, surprise *r* = .32, *p* < .01). Finally, no associations were found between empathic responsiveness and parent reported empathic skills (SRS T1: *r* = − .04, *p* > .05 T2: *r* = .00, *p* > .05, and ToMbc T1: *r* = .13, *p* > .05, T2: *r* = .26, *p* > .05).

**Fig. 2:**
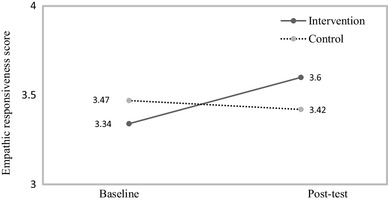
Difference between the two groups in empathic responsiveness (n = 135). Empathic responsiveness score refers to the average combining the two situations and ratings from two raters are

The repeated measures ANOVA testing the effect of training on empathic responsiveness indicated a significant time*group interaction (*F* (1,133) = 4.66, *p* < .05, *η*_*p*_^2^ = 0.03) (see Fig. 2). Post hoc analyses investigating the effect of time separately for the intervention and waitlist control conditions indicated that participants in the intervention condition showed a significant increase in empathic responsiveness (M_baseline_ = 3.34 (SD: 0.79), M_post-test_ = 3.60 (SD: 0.60), *F* (1, 71) = 7.74, *p* < .01, *η*_*p*_^2^ = 0.10), while there was no effect for participants in the control condition (M_baseline_ = 3.47 (SD: 0.72), M_post−test_ = 3.42 (SD: 0.71), *F* (1, 62) = 0.24, *p* = .63, *η*_*p*_^2^ = 0.00). However, no significant differences occur between the two groups when comparing them on empathic responsiveness only at post-test assessment (*F* (1,133) = 2.52, *p* = .115, M_intervention_ = 3.60, (SD: 0.61) [range 2–4.5], M_control_ = 3.42, (SD: 0.71) [range 1–4.5]). Additionally, when testing the association without any missing values, the direction and the nature of the association remained similar Intervention group: M_baseline_ 3.4 (SD: 0.75) and M_post-test_ 3.6 (SD: 0.63), Control group: M_baseline_ 3.5 (SD: 0.72), M_post-test_ 3.4 (SD: 0.65), but did not remain significant (*p* = .11).

Moreover, in the exploratory analysis, comparing the two assessment situations (N = 127), group differences were bordering significance when an experimenter expressed excitement (*F* (1 125) = 3.86, *P* = .052, *η*_*p*_^2^ = 0.03), but no significant differences were found when expressing surprise (*F* (1 125) = 0.07, *P* > .05, *η*_*p*_^2^ = 0.001). Regarding excitement, empathic responsiveness in the intervention group was M_baseline_ = 3.29 (SD: 1.10), M_post-test_ = 3.57 (SD: 0.92), while participants in the control condition scored M_baseline_ = 3.62 (SD: 0.84), M_post-test_ = 3.51 (SD: 0.86). Regarding surprise, empathic responsiveness in the intervention group was M_baseline_ = 3.44 (SD: 0.75), M_post-test_ = 3.58 (SD: 0.79), while participants in the control condition scored M_baseline_ = 3.29 (SD: 0.94), M_post-test_ = 3.39 (SD: 0.77).

## Discussion

This study investigated whether a Theory of Mind training can improve observed empathic responsiveness in youth with ASD by comparing change in empathic responsiveness from baseline to post-test for an intervention and waitlist control group. As hypothesized, the intervention group improved significantly in empathic responsiveness, while the control group did not. This indicates that Theory of Mind training does not only improve ToM understanding and ToM-related behaviour as assessed with child-based measures and parental reports (Begeer et al. 2011, 2015; Fletcher-Watson et al. 2014) but also improves children’s empathic behaviour in naturalistic situations.

The current findings are in line with previous studies on the current trial, which showed an increase in parent-reported ToM related behaviour after a ToM training (i.e. increased scores on the ToM Behaviour Checklist and the Social Cognition subscale of the Social Responsiveness Scale) (Begeer et al. 2015; De Veld et al. 2017). However, previous results also showed that parental reports of children’s general social skills remained unaffected after ToM training (Begeer et al. 2015; Fletcher-Watson et al. 2014). This pattern of results may indicate that the main benefits of ToM training are improvements in empathic behaviour and social understanding, concepts that are explicitly addressed in the training, with little generalization to other aspects of social behaviour. Objective, sensitive and targeted outcome measures may provide a more realistic picture of the effects of ToM interventions on specific ToM related domains of functioning in real life social interactions (Peterson et al. 2016). The realistic picture may also be slightly more optimistic about the effects of ToM interventions, which are currently dismissed as not efficacious in reviews (Marraffa and Araba 2016).

One major strength of this study is the use of structured observations of children’s spontaneous responses to a naturalistic event as a means of assessing empathic responsiveness. The unexpected occurrence of these events in between the remaining psychological tests further strengthens the resemblance to everyday situations, as real life social dynamics are usually unstructured and unexpected, making them challenging for youth with ASD (Baron-Cohen and Wheelwright 2004). As applying skills from the training context to real life is often difficult for children with ASD (Fletcher-Watson and McConachie 2010), such observations may be instrumental in addressing whether taught skills will be used in everyday life. Furthermore, structured observation conducted by blinded assessors may offer more realistic estimates of the intervention effect than parental reports, as parents are aware of whether their child is in the intervention group or in the waitlist group. Finally, similar to previous studies (e.g., Scheeren et al. 2013), structured observations and parent reported empathic skills (SRS, ToMbc) were not found to be associated. This may suggest that parental measures can more likely be influenced by general trait information on the child, while structured observations rely on specific circumstances of the child in the company of an adult stranger. Importantly, both measures offer however important information about the intervention effects.

Although this study is based on a large sample RCT using naturalistic observations, there are also some limitations. The improvement in empathic responsiveness in response to the ToM training was significant but small. When testing the association without any missing data, and thus with a smaller sample size (N = 129), the result did not remain significant. Yet, regardless this non-significant finding, the nature and of the association remained the same, showing an intervention effect. Also, the control group’s performance decreased slightly (but non-significantly) over time, which may have influenced the findings. This decrease may relate to the increased familiarity with the researcher and the testing situation at post-test, but these speculations need future confirmation. The intervention effect could also be due to the lower baseline score in the intervention group. Furthermore, it is possible that behaviour, which was rated as “relevant response”, included also some aspects of “empathic response”.

Small improvements in the intervention group may also relate to empathic behaviour being generally quite rare among youth when interacting with an unfamiliar adult (Scheeren et al. 2013; Newbigin et al. 2016). One direction for future research would thus be to test whether the effect is bigger with a familiar adult. Second, future research could include a broader range of displayed emotions. A previous study used a broader range of emotions displayed by the experimenter and correspondingly reported a broader range of emotional reactions in the children (Scheeren et al. 2013). Including a multitude of emotions could therefore result in more variability in empathic responsiveness, which can be beneficial when aiming to differentiate which children benefit most from training. Third, considering new possibilities to better identify empathic behaviour which is not expressed verbally is another potential direction for future studies.

When comparing the included sample to the participants who were excluded due to missing data, it seemed that the excluded sample scored somewhat lower on verbal intelligence. Thus, the current sample may not have had a representative variation regarding verbal intelligence. This, may limit the generalisability of these findings, as well as the small number of girls, the large number of children with PDDNOS diagnosis and a group of participants (9.2%, n = 12) who did not score above the clinically significant threshold (≥ 59) for ASD on the Social Responsiveness Scale (SRS). Thus, future studies would benefit from the inclusion of more girls and a sample including broader variety of verbal intelligence and ASD diagnoses. Finally, further research is needed to assess the clinical significance of the current, novel measure on empathic responsiveness, especially because it did not associate with the previously validated measures on parent reported empathic skills.

Despite these limitations, this large-sample study contributes to the existing body of knowledge on ASD by investigating the effect of ToM training on the real-life empathic behaviour of children with ASD. When investigating effects of interventions, we face the challenge of finding measures that are adequately focussed on the concept(s) that we aim to change, yet are still generalizable to real life situations. Structured observations can be instrumental in this regard. In the current study, structured observations of empathic behaviour indicate that ToM intervention is not only able to improve ToM related skills and behaviour measured with questionnaires or parental questionnaires but may also improve empathic responsiveness in a naturalistic interaction with unfamiliar adults.
